# Evaluation of off-target and on-target scoring algorithms and integration into the guide RNA selection tool CRISPOR

**DOI:** 10.1186/s13059-016-1012-2

**Published:** 2016-07-05

**Authors:** Maximilian Haeussler, Kai Schönig, Hélène Eckert, Alexis Eschstruth, Joffrey Mianné, Jean-Baptiste Renaud, Sylvie Schneider-Maunoury, Alena Shkumatava, Lydia Teboul, Jim Kent, Jean-Stephane Joly, Jean-Paul Concordet

**Affiliations:** Santa Cruz Genomics Institute, MS CBSE, University of California, 1156 High Street, Santa Cruz, CA 95064 USA; Central Institute of Mental Health, Medical Faculty Mannheim, Heidelberg University, Square J5, Mannheim, 68159 Germany; Institut Curie, CNRS UMR3215, INSERM U934, Paris, Cedex 05 75248 France; CNRS UMR 7622, INSERM U1156, Sorbonne Université Paris 06, Paris, France; Mary Lyon Centre, MRC Harwell, Didcot, UK; TEFOR Infrastructure, Gif-sur-Yvette, France; INSERM U1154, CNRS UMR 7196, Muséum National d’Histoire Naturelle, Paris, France

## Abstract

**Background:**

The success of the CRISPR/Cas9 genome editing technique depends on the choice of the guide RNA sequence, which is facilitated by various websites. Despite the importance and popularity of these algorithms, it is unclear to which extent their predictions are in agreement with actual measurements.

**Results:**

We conduct the first independent evaluation of CRISPR/Cas9 predictions. To this end, we collect data from eight SpCas9 off-target studies and compare them with the sites predicted by popular algorithms. We identify problems in one implementation but found that sequence-based off-target predictions are very reliable, identifying most off-targets with mutation rates superior to 0.1 %, while the number of false positives can be largely reduced with a cutoff on the off-target score. We also evaluate on-target efficiency prediction algorithms against available datasets. The correlation between the predictions and the guide activity varied considerably, especially for zebrafish. Together with novel data from our labs, we find that the optimal on-target efficiency prediction model strongly depends on whether the guide RNA is expressed from a U6 promoter or transcribed in vitro. We further demonstrate that the best predictions can significantly reduce the time spent on guide screening.

**Conclusions:**

To make these guidelines easily accessible to anyone planning a CRISPR genome editing experiment, we built a new website (http://crispor.org) that predicts off-targets and helps select and clone efficient guide sequences for more than 120 genomes using different Cas9 proteins and the eight efficiency scoring systems evaluated here.

**Electronic supplementary material:**

The online version of this article (doi:10.1186/s13059-016-1012-2) contains supplementary material, which is available to authorized users.

## Background

The CRISPR/Cas9 “revolution” [1] is sweeping through the life sciences. As more researchers face the task of selecting an optimal Cas9 guide RNA sequence that targets a genome sequence of interest, the overall specificity of the technique is still under discussion: high-throughput cell culture studies have found numerous off-targets not predicted by existing algorithms, sometimes even involving 1-bp indels (“bulges”) in the alignment with the guide sequence [2, 3], while studies in *Drosophila*, *Caenorhabditis elegans*, zebrafish, and mice have found virtually no off-target effects [4–6]. The guide sequence also determines the efficiency of on-target cleavage [7, 8]; thus, current genome editing protocols recommend [9] that researchers select guides carefully to minimize potential off-target effects and test several to optimize on-target activity. Although published tools and scoring systems allow ranking sequences by specificity [10–22] and efficiency [2, 8, 23–25], they are usually limited to a handful of genomes and only few evidence-based recommendations exist to optimize off-target search parameters and on-target efficiency. In this article, we compare existing scoring systems against published datasets and our own experimental data. The optimal selection parameters that we identified were integrated into a new CRISPR/Cas9 guide designer tool.

## Results and discussion

We developed a novel web-based tool, CRISPOR (http://crispor.org), to assist with guide selection in 120 genomes, including plants and many emerging model organisms, and pre-calculated results for all human coding exons as a UCSC Genome Browser track. To evaluate off-target prediction accuracy, we took advantage of eight recently published studies that detected and quantified off-target cleavage sites [2, 3, 7, 26–29] (summarized in Additional file 1: Table S1) and from these collected 650 off-target sequences that were experimentally identified for 31 different guides (Additional file 2). The assays differed mostly in sensitivity (Additional file 3: Figure S1; Additional file 4: Table S2, and Additional file 5: Table S3). Two studies [3, 28] did not validate identified off-targets with PCR amplicon sequencing in the same cell type and may include false positives.

We noticed two outliers, VEGFA_site2 and HEK293_sgRNA4, from the study by Tsai et al. [3]. The two guides are responsible for 151 and 133 off-targets, respectively. Together they account for 44 % (284/650) of all off-target sequences in our dataset and 71 % (84/119) of the off-targets with five or more mismatches. They also have the highest GC content in the Tsai et al. data, 80 % and 75 %, respectively (Additional file 6: Figure S2). A relationship between GC content and specificity is known from siRNA design [30] and would explain the previously observed difficulty to target GC-rich genes [2, 8, 31] and quadruplex-forming sequences [32]. Of all four million unique -NGG guide sequences in human coding exons, the ones with a GC content >75 % constitute only 13 %, so they can usually be avoided. We therefore removed these two guides from further analysis.

One issue with the remaining data was the sensitivity of the assays. The two assays using targeted sequencing of predicted sites reported off-targets with a modification frequency lower than 0.001 % [7, 29] while all whole-genome assays estimated their sensitivity at around 0.1–0.2 % [2, 3, 33] (Additional file 1: Table S1; [26, 28] did not report sensitivity). This means that the rare off-targets found in targeted sequencing studies cannot be compared with those from whole-genome assays. We therefore chose to analyze only off-targets that can be detected with whole-genome assays, with a modification frequency >0.1 %.

**Fig. 1:**
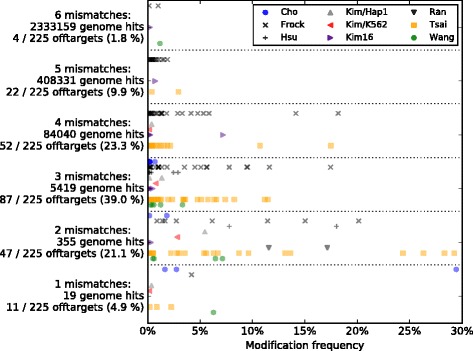
The 225 off-target modification frequencies for 26 guide RNAs separated by number of mismatches. To indicate the optimal depth for an off-target search, validated off-target modification frequencies are shown on the *x-axis*, separated by number of mismatches from their guide sequence (*rows* along the *y-axis*). The studies are indicated by *symbols*, explained in the legend of the graph [3, 7, 26, 28, 29, 33, 38]. The *row label* specifies the number of mismatches, followed by a *line* showing the total number of off-targets predicted by CRISPOR for the 22 guide sequences (“genome hits”). The *third line* indicates the number of validated off-targets and the percentage of total validated off-targets that they represent. For example, at six mismatches, about 1.9 million potential off-targets were found in the genome for the 26 guide sequences, three of which were shown to be bona fide off-targets that were experimentally validated. The four off-targets with six mismatches make up only 1.8 % of all off-targets, so 98.2 % of the 225 off-targets differ by up to five mismatches. The off-targets with five and six mismatches make up 11.7 % of all off-targets

Of the remaining 225 off-targets, most (88.4 %) had up to four mismatches relative to the guide (Fig. 1). All others had five or six mismatches but with low modification frequencies, <3 % or <1.1 %, respectively. Most of these were found by Frock et al. [28], a study that seems to favor more degenerate off-targets and did not validate them with PCR. Allowing indels (“bulges”) in the alignment would have made a difference only for two off-targets out of 225, with cleavage frequencies of 0.1 % and 0.2 %, as previously observed [2, 28] (Additional file 1). In addition, the ranking of the guides by MIT specificity score (see below) was largely unchanged when increasing the number of mismatches beyond four (Additional file 7: Figure S3). Therefore, CRISPOR does not allow indels, ranks guides based on potential off-targets with up to four mismatches, and allows five mismatches for a detailed analysis of a single guide.

It has been reported that the off-target predictors on the CRISPR Design website (http://crispr.mit.edu) and Ecrisp [10] failed to detect many off-target sites [3, 34], including off-targets with a single mismatch from the guide. In contrast, we confirmed that the BWA [35] sequence search algorithm used in CRISPOR as well as the novel algorithm in the recently published CasOffFinder [11] were able to find all validated off-targets (Additional file 8: Table S4), demonstrating that this is only a software issue and limited to certain tools. For example, in the case of the EMX1 guide, CRISPOR and CasOffFinder predict 1288 off-targets with up to four mismatches while the MIT site predicts only 334 and as a result does not find five out of 15 validated off-targets, one of which has only two mismatches and a >20 % modification frequency confirmed by two different assays (Additional file 4: Table S2 and Additional file 5: Table S3).

In order to rank potential off-targets, many prediction tools calculate a score based on the position of the mismatches to the guide sequence. Initially, systematic testing of the effect of mismatches led to a weight for each possible nucleotide change at each position and a formula to combine these into a score [7]. The score of the MIT website (http://crispr.mit.edu/about) is based on these data but reduced to one weight per position. The off-target predictors CCTop [36] and CROP-IT [37] independently devised heuristics based on the distances of the mismatches to the protospacer adjacent motif (PAM). The more recent CFD score [34] is based on the biggest dataset to date, cleavage data obtained by infecting cells with a lentiviral library containing thousands of guides targeting the CD33 gene for all PAMs, including guides for all possible nucleotide mismatches and 1-bp indels at all positions. In addition, all scores except CCTop also include a penalty for mismatches located close to each other.

**Fig. 2:**
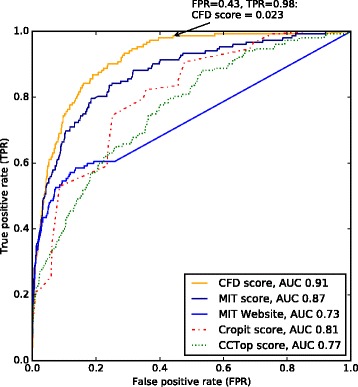
Receiver operating characteristic of CRISPOR using various off-target scores and versus the CRISPR Design website (http://crispr.mit.edu/). We used 26,034 putative off-targets identified by CRISPOR as the elements classified by the tools. The MIT website has a search depth of four mismatches, so off-targets with more than four mismatches were not considered for this graph. *MIT score* refers to the MIT off-target score as calculated by the CRISPOR website, *MIT Website* refers to the MIT off-target score as calculated by the CRISPR Design website (http://crispr.mit.edu/). For each scoring method, shown are the True positive rate (*TPR*)/False positive rate (*FPR*) when classifying 143 validated off-targets with a mismatch count of up to four, one of the PAMs NAG/NGA/NGG, and a minimum modification frequency of 0.1 %. The *arrow* marks the performance when using a CFD score cutoff value of 0.023. It leads to a 98 % true positive rate and a false positive rate of 43 %

For off-targets with up to four mismatches, receiver-operating characteristic analysis (ROC; Fig. 2) of these four algorithms shows that the CFD score distinguishes best between validated and false-positive off-targets, with an area under the curve (AUC) of 0.91. The MIT score as calculated by the CRISPOR website is slightly less discriminative with an AUC of 0.87. As expected, when calculated by the MIT site itself, the AUC of the MIT score is a lot lower because this tool misses many off-target alignments in the genome. The ROC plot also shows that adding a minimal CFD off-target score of 0.023 decreases false positives by 57 % while reducing true positives by only 2 %. At this cutoff, no off-targets with a modification frequency >1 % are missed (data not shown).

**Fig. 3:**
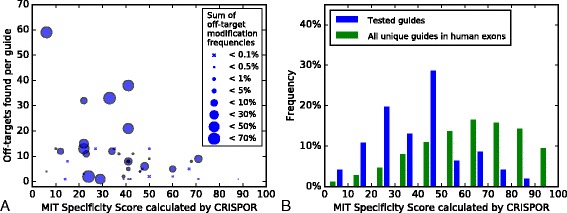
Cross-study analysis of MIT specificity scores as calculated by CRISPOR versus the number and strength of off-targets found. **a** For 31 guide sequences, CRISPOR guide specificity scores are shown (*x-axis*), as well as the number of off-targets (*y-axis*) and sum of off-target modification frequencies (*circle size*). The guide with a specificity score of 88 has no single detected off-target. **b** The specificity scores of 31 tested guide sequences (*blue*) versus the specificity scores of all unique guides (unique 20mers followed by NGG) in human coding regions (*green*). Specificity scores were calculated using the CRISPOR website. For a version of this figure with specificity scores calculated by the MIT site, see Additional file 9: Figure S4

We next examined the ranking of guides by specificity. The MIT scores of all potential off-targets of a guide can be summarized into the “guide specificity score” defined by [7], which ranges from 0–100 (100 = best). Figure 3a shows that higher specificity scores are generally associated with fewer off-target sites and lower off-target modification frequencies, as expected. In contrast, a few guides had unusually strong off-targets, illustrating that the scoring model could still be improved, possibly by using the CFD off-target score or taking into account the chromatin context [3, 7]. However, a single score for guide specificity may not always be valuable. For example, intergenic off-targets may be considered a minor issue for functional studies in cultured cells. When transgenic animals are back-crossed, off-targets on a different chromosome will not co-segregate with the mutation of interest and may often be acceptable. Therefore, while CRISPOR shows the MIT specificity score as an indicator of guide quality, all potential off-targets are annotated and shown for detailed inspection.

We ranked the four million unique guide sequences in human coding regions by MIT specificity score. We observed that the guides tested in the eight off-target studies exhibit relatively low specificity scores relative to the genome average (Fig. 3b). The relatively low specificity scores make the high number of off-targets that were found less surprising. As a result, there is currently limited data on guides with high specificity scores that are more relevant when designing an experiment. Figure 3b shows that the more specific guide RNAs that were tested as well as about 30 % of the guide sequences in human coding regions exceed a specificity score of 50. Therefore, the CRISPOR website highlights guides with a minimum MIT specificity score of 50. With the MIT website, as it misses some off-targets, the cutoff should be higher, around 70–80 (Additional file 9: Figure S4).

In addition to off-target cleavage, we evaluated predictions of on-target efficiency, including eight different scoring models and two heuristics. For this purpose, we collected activity data for more than 19,000 guides, including data sets used to build the scoring models [6, 8, 23–25, 34, 38, 39] and from independent studies in cultured cells and ascidian oocytes and from zebrafish screens [31, 40–43]. Additional file 10: Table S5 summarizes the studies and the different assay types.

For datasets where replicates are available, the Spearman correlation is in the range 0.71–0.77 (Additional file 11: Table S6; Hct116, mouse embryonic stem cells) for the same assay in the same cell type. This gives an indication of the quality of the data and suggests that a correlation of about 0.7 constitutes an upper limit of any prediction. For some datasets, the assay was repeated in a different cell type. In these cases, the correlations were almost identical for some cell type combinations (e.g., 0.75 for Hl60/Kbm7 [38]; Additional file 11: Table S6) and lower for others (0.53–0.63 for Rpe1 cells [41]). If these lower correlations are due to differences in the chromatin state, this suggests that its influence varies and is relatively modest, at most 10–20 % of the rank correlation.

**Fig. 4:**
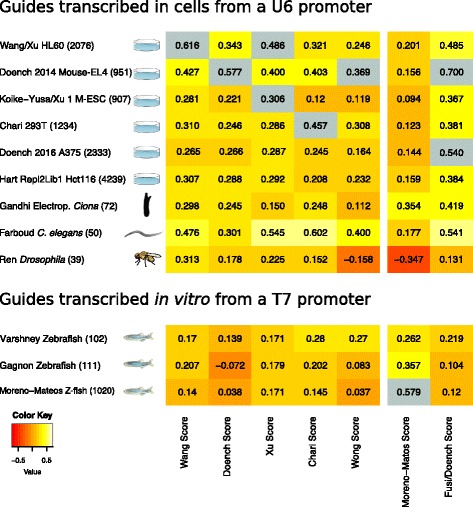
Heat map of Spearman rank correlation coefficients between efficiency scores and datasets. For each dataset, the experimental system is indicated by a species icon or cell type. Number of guides tested are shown in *parentheses*. Scores are shown along the horizontal axis, datasets on the vertical. Correlations of an algorithm against its own training dataset are shown in *grey* as they are likely to be overestimated due to overfitting. The datasets Wang/Xu HL60 and Koike-Yusa/Xu on mouse embryonic stem cells are originally from Wang et al. [38] and Koike-Yusa et al. [54] but were used as processed by Xu et al. [23]. From the dataset by Hart et al. [41], only the cell line Hct116/repeat2 was used, as it gave the highest correlation value; for this study efficiency was averaged over all time points. Data on human cell lines for the two datasets by Doench et al. [8, 34] are not shown here but gave an almost identical correlation profile. All data points are shown as scatter plots in Additional file 12; for assay background information on the datasets see Additional file 8: Table S4

The heat map in Fig. 4 shows that on independent datasets, those not used to train any algorithm such as Hart et al. [41], current predictions achieve Spearman correlations of 0.341–0.436 (see Additional file 12 for plots of individual data points). In cases when algorithms are applied to their own training dataset the correlations are higher, but this is an artifact, known as algorithmic overfitting; we show the corresponding correlation values in grey in Fig. 4.

We observed that the quality of the assay is an important parameter. For example, for a dataset obtained with Surveyor Nuclease, we found no significant correlation between guide activity and any of the scores (see “Liu” in Additional file 13: Figure S5). Another example is the Housden et al. score [44], which did not predict well the activity in any dataset, including its own. This may be due again to the accuracy of the activity measurements or a result of the statistical model used by Housden et al., a weight matrix. The dataset “Wang 2015” was designed with a scoring algorithm and shows very little signal. The dataset “Eschstruth” is very small and includes several guides that were selected based on very high Doench scores. In the Chari et al. study [24], the dataset from K562 cells was not correlated with two replicates of the same assay in HEK293T cells, so we only used the HEK293T dataset, like Chari et al. themselves. We do not show these five datasets in Fig. 4 but instead in Additional file 13: Figure S5; the raw data are included in Additional file 14.

Figure 4 shows that scores trained on mammalian cell lines work surprisingly well in other organisms, even in non-vertebrate ones, like *Ciona intestinalis*, *C. elegans*, and, to some extent, *Drosophila*, though in the latter only limited data are available. In contrast, the Moreno-Mateos score, an algorithm trained on zebrafish assays, does not translate well to all other datasets and vice versa. This is consistent with previous reports that the Doench score is not accurate in zebrafish [31, 40]. For this organism, guides are made by in vitro transcription with the T7 promoter and injected into eggs rather than expressed from exogenous DNA in cells from a U6 promoter. Without constant expression of the guide from a plasmid, the stability of guide RNA starts to play a bigger role [39]. Possible explanations for the difference in algorithm performance are, therefore, that RNA stability or the promoter leads to differences in guide activity. By excluding artifacts (*grey*) in Fig. 4 and taking this separation into account, one can hypothesize that the Fusi/Doench score performs best in U6 promoter-based assays and Moreno-Mateos best in assays based on delivery of guide RNAs produced by T7 in vitro transcription.

**Fig. 5:**
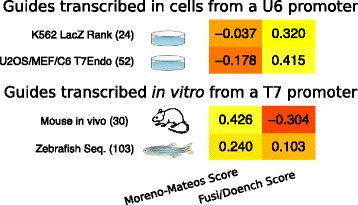
Heat map of Spearman rank correlation coefficients for the best two efficiency scores from Fig. 4 and four novel datasets from this study. Correlations are shown as in Fig. 4

To confirm this observation and to rule out an influence of the organism or the assay itself, we analyzed data from our own labs in the same way (Fig. 5). We tested two series of guides in cell cultures with two different assays (“K562-lacZ rank” and “U20S/MEF/C6-T7 endo”, 24 and 49 guides, respectively), injected one series of guides in zebrafish one-cell embryos (“Zebrafish-seq”, 163 guides) and another series in mouse embryos (“Mouse in vivo Seq”, 30 guides) (Additional file 15: Table S7). The data confirmed that zebrafish and cell culture results differ and most importantly they showed that the mouse in vivo data, using in vitro transcribed guide RNA, correlates best with the zebrafish-based predictor (Spearman *P* value 0.019; see Additional file 12 for *P* values and Additional file 14 for all frequencies and prediction scores where these data sets are called “Schoenig”, “Concordet”, “Shkumatava”, and “Teboul”, respectively).

Correlation of the prediction score with observed absolute activity may disadvantage some algorithms. We therefore performed precision-recall curve analysis (Additional file 16: Figure S6) and also calculated precision/recall based on the overlap of the top quartile of the predictions with the top quartile of measured activity (Additional file 17: Figure S7). For the latter, we added two heuristics described by [6, 25], GC content in the last four base pairs and whether the guide ends with -GG. The results overall correspond to the performance as measured by correlation values; the Fusi/Doench and Moreno-Mateos scores perform best on the large datasets and depend on the expression system.

Two prediction schemes can reach a relatively high precision: the Wong score [45] for cell cultures (U6 promoter) and the -GG rule for T7 in vitro transcription. However, their recall is relatively low; in the Doench 2014 dataset, for example, only 12.8 % of guides have a Wong score that is not zero and 13.2 % end with -GG.

CRISPOR calculates all currently available scores and lets the user select the most suitable one for the particular assay/model organism. Based on Fig. 4, we recommend the Fusi/Doench score for guides expressed from a U6 promoter and the Moreno-Mateos score for experiments where guides are produced by T7 in vitro transcription. As an additional ranking criterion, when there is a large set of possible guides to pick from, the Wong score and -GG rule predict well efficient U6- and in vitro-transcribed guides, respectively.

Are correlations of around 0.4 high enough to reduce the number of guides in practice? To demonstrate that the efficiency scores are useful not only when designing thousands of guides for genome-wide screens [38] but also in a more common genome editing project of just a few loci, we evaluated the prediction performance on the data from our labs shown in Fig. 5. For two datasets, we have screened multiple guides per locus to select the most efficient one and evaluated post hoc how much time could have been saved by using the appropriate prediction algorithm.

In the K562 cell culture dataset, three guides each from eight loci in human, mouse, and rat were tested with an in vitro assay [46] (dataset “Schönig” in Additional file 14). For six out of eight loci, the highest Fusi/Doench score did predict the guide with the strongest cleavage (*P* = 0.032). In another set of 104 guides from 11 zebrafish loci (“Shkumatava” in Additional file 14), taking only two guides with the highest Moreno-Mateos score from each locus would have reduced the number of injections from 104 to 22 and still identified one of the top two guides for nine out of 11 loci (*P* = 0.024; no other score was significant). In both cases, a second round of screening would have been required, but the number of guides to screen could have been reduced by a third. In the case of the zebrafish screen, which are typically more time-consuming than cell culture assays, we estimate that we could have saved 250 h of work by using the Moreno-Mateos score. In addition and especially in mice, the ability of predicting guide RNA activity is a significant advance in terms of animal welfare as fewer animals will be required to create mutants.

## Conclusions

Our collection of off-target sites confirms that, overall and across all studies, bulges are rare and extremely GC-rich guides should rather be avoided. For the remaining sites, sequence-based prediction performance has to be seen relative to the sensitivity of the experimental system used to validate the off-targets. When using a cutoff on the CFD score, predictions contain 98 % of off-target sites validated by whole-genome assays (sensitivities > ~0.1 %), with a 43 % false positive rate. As targeted sequencing is the most sensitive assay for most applications, predicting off-target sites and validating them with targeted sequencing seem easier and more sensitive than any of the whole-genome off-target assays like Guide-Seq and Digenome-Seq. However, sites have to be predicted with a software package that reliably identifies sites with at least four mismatches, like CRISPOR or CasOffFinder, not the current versions of the CRISPR Design website (http://crispr.mit.edu/) or Ecrisp.

Our comparison of on-target activity predictions confirms that they can significantly reduce the effort spent on screening guides, but we found that the prediction model trained on data from the same guide expression system (U6 versus T7 in vitro transcription) has to be used. In particular, Figs. 4 and 5 indicate that the results of guide injections into mouse oocytes, the most expensive experiment in this field, despite being the organism where thousands of cell culture data points are available, are currently best predicted by an algorithm trained on injection results from a non-mammalian organism, zebrafish.

Our summary of all publicly available data, the predicted scores, and source code to calculate these should simplify future computational work on CRISPR/Cas9 off- and on-target predictions. For wet lab experimentalists who want to integrate the current state of the art into their experimental design, our website (http://crispor.org) includes pre-calculated results for all human exons on the UCSC Genome Browser tracks and can calculate off-target scores, all efficiency scores, CG content warnings, score cutoffs as presented in this article, and PCR primers in 120 genomes within minutes for any sequence of interest. We hope that the results and resources presented here will aid with future improvements and wider adoption of CRISPR/Cas9 off- and on-target prediction algorithms and reduce the time spent on screening for off-targets and efficient guide sequences.

## Methods

### Individual off-target datasets and modification frequency

We obtained lists of guide sequences and their off-targets from studies [3, 7, 26–29] that tested 20-bp-long guide sequences. Data were extracted from supplemental files with the PDF table extraction software Tabula (http://tabula.technology/).

In the case of a study that tested both 19- and 20-bp guides [33], for consistency we used only the 20-bp guide data but included data from both cell lines (HAP1 and K562). For all studies, we obtained a measure of cleavage, the “modification frequency”, the number of all successful genome insertions or deletions divided by all observations at the respective off-target site, as reported by [7] and [29]. For a study that quantified modifications using both sequencing and lentiviral insertions [38], we did not use the low-resolution number of lentiviral insertion sites but rather the frequencies from targeted sequencing, which the authors kindly shared with us. Tsai et al. [3] measured only successful modification events, so as an approximation of modification frequency we divided reads per site by all reads obtained for one guide. Two studies [3, 28] observe only modifications, so the sum of the frequencies of a single guide is always 1.0, which is not the case for the other datasets. Frock et al. [28] did not directly quantify genomic insertions or deletions but counted the correlated events “lentiviral insertion”, which samples relatively rare cleavage events and may as a result overestimate real modification frequencies.

The complete dataset consists of 30 guide sequences tested by 36 assays, 634 off-target sequences, and 697 cleavage frequencies, as some off-targets were detected by different assays. For an overview of all off-target studies see Additional file 1: Table S1; for the complete off-target dataset see Additional file 2.

### Cleaning the off-target datasets

After removal of the two GC-rich guides and 0.01 % modification frequency filtering, the filtered dataset contained 225 modification frequency measurements of 179 off-target sequences for 31 tested guide sequences, of which 26 guide sequences contain off-targets >0.01 %.

### Off-target scores

From the description in the article [7] there are several possibilities to calculate the Hsu score; we used only the normalized aggregate frequencies which also gave the highest AUC. The off-target and specificity score of the MIT website were implemented based on source code by Hari Jay (https://snipt.net/harijay/fz-score-d1324dab/). We implemented the CROP-IT and CCTop off-target scores from the description in the original articles [36, 37]. For the CFD score [34], we received source code from the authors.

### Previously published knock-out and cleavage efficiency datasets

The efficiency studies are summarized in Additional file 10: Table S5. We used the human knock-out efficiency dataset from Wang et al. as provided by Xu et al. [23, 38] for HL60 cells, inversing the sign, such that higher values mean a more efficient knock-out, as in the other studies. The dataset by Doench et al. [8] was used as rank-percent values as provided; we also converted parts of the raw data to log-abundance values, as described in their study, and split them by exon and cell type. For the newer dataset from Doench et al. [34], we used their Supplemental Table 16 and only the eight genes with reproducible results across the treatments (CCDC101, CUL3, HPRT1, MED12, NF1, NF2, TADA1, TADA2B) as recommended in their study. Guides that did not uniquely map to the human genome (hg19) were removed, resulting in 2333 guides. For Chari et al. [24], we used only the *Streptococcus pyogenes* dataset from 293 T cells, as the K562 dataset was not correlated with any score nor their 293 T results. Datasets from [6, 25, 31, 40, 42, 43] were used as provided. At first we did not obtain any significant correlation for the dataset by Housden et al. [44] and after notifying the authors and received a corrected version of their Additional file 1: Table S1. For the dataset by Hart et al. [41], we received the log-fold changes in five different cell lines and time points 8–18 days from the authors. The guides in this study were selected to have a GC content in the range 45–70 % and no T in the last 4 bp. We kept only data for 4293 guides against the 829 genes determined to be essential by the authors in all five cell lines, used the average over all time points as the assay result, and used only the result from the Hct116, library 1, replicate 1 which was the only cell line with a replicate and with a high correlation between both replicates and the focus of the original study (Additional file 10: Table S5). A very recent CRISPR library [47] was designed using the Wang score and, due to this bias, is not usable for our evaluation.

All sequences without genomic coordinates were mapped with BLAT [48] to the respective genome and extended by 50 bp on both sides of the protospacer adjacent motif (PAM) to provide enough flanking sequences for the score calculations. For the Doench 2014 dataset, duplicate genomic hits were resolved manually to a single hit. For the other datasets, guides with duplicate matches were skipped. All tested guide sequences and their reported efficiencies are available in Additional file 12.

### New cleavage efficiency datasets

Our first cell culture dataset (“Schönig” in Additional file 14 and Additional file 10: Table S5) is a set of 24 guide sequences, three guides each from eight loci, one locus in human, five in rats, two in mice, tested with a lacZ nuclease activity assay [46]. For this purpose, the guide RNA target regions were inserted into a nuclease reporter plasmid (pTAL-Rep^37^) in between a partly duplicated, nonfunctional β-galactosidase gene. Upon transfection of the reporter plasmid and px330-U6-based guide RNA expression vectors [49] into HeLa cells, nuclease-induced double-strand breaks stimulate the repair of the gene segments into a functional reporter gene, the activity of which is determined in cell lysates using an o-nitrophenyl-β-D-galactopyranosid (ONPG) assay. A luciferase expression vector was also added to the transfection mix and luciferase activity was measured as transfection control. Each sample activity was ranked from 1 to 3, with 3 representing strongest cleavage. The *P* value in the text is the probability to obtain six or more guides with an activity of 3 when randomly drawing one guide per locus and repeating the sampling 100,000 times.

The second cell culture dataset (“Concordet” in Additional file 14 and Additional file 10: Table S5) was obtained from 52 guide sequences targeting 14 loci. The guides were cloned into the MLM3636 plasmid with a U6 promoter (Addgene #43860, from the KJ Joung lab), and electroporated into cells. PCR products were tested with the T7 endonuclease assay [50] and mutated sequences quantified by gel electrophoresis. The result of the T7 assay was reported as the numbers 1, 2, or 3 based on the mutation rate: 1, inactive guides; 2, not very active guides; 3, efficient guides (mutation rate > 10 %). In total, 26 guides from the human genome were tested in U2OS cells, 18 mouse guides in mouse embryonic fibroblast (MEF) cells, and eight rat guides in C6 cells.

Our first zebrafish dataset (“Eschstruth” in Additional file 14) is a set of 18 guide sequences targeting a single locus in zebrafish. Three of the guides were selected because of their high Doench scores. We injected 20–50 pg gRNA transcribed from a T7 promoter into zebrafish one-cell embryos with 300 pg Cas9 mRNA. Cleavage efficiency was measured on 16 single embryos 24 h post-fertilization with the T7 assay, a standard protocol described previously [50] and classified into three categories: no cleavage (1), low cleavage (2), and high cleavage (3). This dataset is shown in Additional file 16: Figure S6, but not Fig. 5 as it is small and the extremely high Doench scores (top 3 %) were used to select the guide sequences, so it is biased compared with the other datasets from this study, where guides were selected without using any predictions.

Our second zebrafish dataset (“Shkumatava” in Additional file 14 and Additional file 10: Table S5) is a set of 103 guide sequences from 11 different loci in zebrafish. Guides were transcribed in vitro with the T7 RNA polymerase kit. No guide was selected based on efficiency scores. Guide RNA (10 pg) and 150–200 pg of Cas9 mRNA were injected into wild-type AB zebrafish at the one-cell stage. Cleavage efficiency was measured by extracting genomic DNA from around 20 embryos, PCR of the target regions, cloning the result into a TOPO-vector, and shipping for Sanger sequencing a number of colonies in the range 10–20. The result of the assay is the number of sequences with mutations over all sequences. Guides were manually assigned to a locus if they were located closer together than 3 kbp. The *P* value in the text is the probability to obtain at least nine successes, where a success is defined as finding at least one guide with a modification frequency among the top two values in a locus when selecting two guides randomly from each locus. The sampling was repeated 100,000 times.

For our mouse in vivo dataset of 30 guides (“Teboul in vivo” in Additional file 14), single guide RNAs (sgRNAs) were synthesized using a MEGAshortscript T7 Transcription kit (Ambion). RNAs were purified using a MEGAclear kit (Ambion). RNA quality was assessed using a NanoDrop (Thermo Scientific) and by electrophoresis on 2 % agarose gel containing ethidium bromide (Fisher Scientific). Cas9 mRNA (5meC, Psi) was commercially purchased (tebu-bio, L-6125-100). Pronuclear microinjection was performed as previously described [51], employing a FemtoJet (Eppendorf) and C57BL/6 N or C57BL/6 J embryos. Cas9 mRNA and sgRNAs were diluted and mixed in MIB to working concentrations of 100 ng/μl and 50 ng/μl each, respectively. For sessions where needles clogged up consistently, the microinjection mix was further diluted with MIB. Injected embryos were re-implanted in CD1 pseudo-pregnant females. Host females were allowed to litter and rear F0 progeny. Genomic DNA from F0 and F1 animals was extracted from ear clip biopsies using the DNA Extract All Reagents Kit (Applied Biosystems). The targeted region was PCR amplified using high fidelity Expand Long Range dNTPack (Roche). PCR products were further purified using a gel extraction kit (Qiagen) and analyzed by Sanger sequencing (SourceBioscience). In total, 496 embryos were tested, 160 of which were mutant. For the absolute counts of embryos for each guide, see Additional file 15: Table S7.

Additional file 17: Figure S7 showns that precision/recall analysis overall gives the same resultsesults as the analysis of Spearman rank correlations. Some scores have a tendency towards higher recall (Fusi, Ren), some towards precision (Wong, Farboud -GG rule).

### Scoring functions

Some of the original articles did not include source code. We implemented the efficiency score by Doench et al. [8] and shared it with Doench et al., who made it available on their website. As a result, our source code has already been used in a study by Xu et al. [23] for an evaluation of the Doench score. We also had to re-implement the Moreno-Mateos score based on the description in the article [39], as the authors declined to share code.

R code to calculate their scoring function was gratefully provided by Wang et al. [38]. We subtracted the result from 1.0 such that higher scores correspond to a better knock out, like all other scores. For the CRISPOR website and better performance, we had to re-implement this score. Even though we are using the same SVM library (libsvm) via scikit-learn [52], the results are slightly different (Pearson R = 0.97, 85 % of the differences are <0.1); the analysis in this article is based on the original R code. Housden et al. gratefully provided Java source code, which we translated it to Python for easier integration into our website.

To obtain Fusi et al. [53] scores, we used the web service at https://www.microsoft.com/en-us/research/project/azimuth/ an API-key gratefully provided by the authors. For a fair comparison with the other algorithms, we did not specify the optional parameter, the position of the guide within the gene. The source code for the Wong et al. score [45] was obtained from the WU-CRISPR website (http://crispr.wustl.edu/) and slightly modified to allow parallel processing of input files.

All efficiency score calculations have been bundled into a Python library (crisporEffScores.py) available from the Github repository accompanying this article (see below). The module includes compiled third-party libraries and their source code: the Xu et al. score and the SVMlight and libSVM libraries that are the basis for the Chari et al. and Wang et al. scores. We hope that authors of new efficiency scores add their code to this module for easier evaluation and integration into future guide selector websites.

To evaluate other off-target predictors, we wrote scripts that automated the web browser Firefox and pasted the guides individually into the CRISPR Design website (http://crispr.mit.edu) and downloaded the results, in total roughly 12,000 predicted off-targets.

### Tool implementation and source code availability

CRISPOR uses the popular BWA aligner [35] version 0.7.5a-r405 in iterative mode (“-N”). All genomic hits within a certain edit distance are retrieved from BWA, filtered for the requested PAM sequence, and scored and annotated with gene model information using the UCSC Genome Browser command line tools [48]. CRISPOR ignores off-targets with an off-target score <0.1 for the PAM NGG and those with a score <1.0 for the PAMs NAG and NGA (Fig. 2). Based on all off-target scores for a guide, a specificity score is calculated using the same formula as on the CRISPR Design website (http://crispr.mit.edu).

CRISPOR currently supports 113 genomes. Potential off-targets can be filtered to retain only those in exons, those that may be of concern when isolating cell clones, or those located on the same chromosome as the target, whose mutations may co-segregate and, therefore, confound phenotypic analysis when studying genetically modified organisms. The predicted guides and their off-targets are shown as a table, with links to the Ensembl and UCSC genome browsers. Results can be downloaded as spreadsheet files for archiving. Several features of practical interest are included, such as primer sequences for cloning into Addgene plasmids, direct expression with T7 RNA polymerase, or PCR amplification of the genome sequence targeted for T7 assays.

All scoring functions have been devised for *S. pyogenes* Cas9 only. Pending further experimental investigation, we have applied scores to engineered *S. pyogenes* Cas9 as well as to other Cas9 proteins shown to work in mammalian cells. The VQR Cas9 mutant was chosen because it discriminates best between NGA and NGG PAMs. The CRISPOR specificity score, similar to the situation with wild-type Cas9, was therefore calculated by ignoring off-targets with an off-target score <0.1 for the PAM NAG and those with a score <1.0 for the PAMs NGG and NGA.

## Additional files

Additional file 1: Table S1. Detailed information about CRISPR/Cas9 off-target studies, including first author, PMID, name of the primary assay, type of genome change detected, cell type, sensitivity, number of guides studied, off-targets found, and whether the off-targets found with the primary assay where subsequently validated by PCR and sequencing. (XLS 36 kb) Additional file 2: Collection of all off-targets and their frequencies from all off-target studies. The fields of the table are: name of the guide (*guide*), its sequence (*guideSeq*), the sequence of the off-target (*otSeq*), the specificity score of the guide determined with up to four mismatches (*guideSpecScore4MM*), the GC content of the guide (*guideGcCont*), the fraction of reads or lentiviral integration sites for this off-target (*readFraction*), the number of mismatches of the off-target to the guide (*mismatches*), the Hsu et al. off-target score (*outscore*), a string indicating with *stars* where the mismatches are located over the length of the guide sequence (*otLogo*), the minimal number of mismatches if a deletion is allowed in the guide sequence (*bulgeRnaMmCount*), the guide sequence with this deletion marked in *parentheses* (*bulgeRnaGuideSeq*), the minimal number of mismatches if a deletion is allowed in the off-target sequence (*bulgeDnaMmCount*), and the off-target sequence with the deletion marked (*bulgeDnaOtSeq*). Bulge information is shown only if the number of mismatches obtained by the deletion is at least three less than the number of mismatches without the deletion; otherwise the fields are set to “−1”. (TSV 98 kb) Additional file 3: Figure S1. Overlap of off-target detection for the EMX1 and VEGFA guides tested by different assays. Off-targets are only shown if they were detected by at least a single study and with a frequency of 0.1 %. See Additional file 1: Tables S1 and Additional file 4: Table S2 for the modification frequencies and additional details on the off-targets for the guides *EMX1* and *VEGFA*, respectively. Additional file 4: Table S2 also includes the data by Hsu et al. [7], who quantified cleavage at putative off-target loci predicted by the CRISPR Design website (http://crispr.mit.edu/) with targeted deep sequencing, Tsai et al. [3], who isolated double-strand breaks with modified oligonucleotides followed by sequencing, Frock et al. [28], who detected translocations, and Kim et al. [33] and Kim et al. [27], who performed whole-genome sequencing to find CRISPR-induced modifications. For details on the different studies, see Additional file 1: Table S1. (PDF 17 kb) Additional file 4: Table S2. Reproducibility of the guide EMX1 GAGTCCGAGCAGAAGAAGAAGGG across three different assays. Shown are the numbers and locations of the mismatches, the off-target score, modification frequencies found by the different studies, and whether the off-target was predicted by the CRISPR Design website (http://crispr.mit.edu/). (XLS 31 kb) Additional file 5: Table S3. Reproducibility of the guide VEGFA GGGTGGGGGGAGTTTGCTCCTGG across three different assays. Shown are the numbers and locations of the mismatches, the off-target score, the modification frequencies found by the different studies, and whether the off-target was predicted by the CRISPR Design website (http://crispr.mit.edu/). (XLS 45 kb) Additional file 6: Figure S2. Ratio of off-target to on-target cleavage for validated guide sequences. The two guides RAG1B and RAG1A are not shown on this plot as their on-target cleavage was not determined in the study by Frock et al. [28]. Studies in the legend are referenced by the first author’s name; in the case of Kim et al., the cell type is also indicated. For each guide, the sum of all off-target modification frequencies was divided by the on-target modification frequency, e.g., a ratio of 2 indicates that cleavage is twice as frequent on all off-targets taken together than on the target. To better show the two outliers, a portion of the *x-axis* and *y-axis* was cut out. The CRISPOR website and Genome Browser tracks show a warning message for guide sequences with a GC content >75 %. (PDF 66 kb) Additional file 7: Figure S3. MIT specificity scores calculated by the CRISPOR website for 28 guide sequences calculated based on predicted off-targets with up to four, five, and six mismatches. Only one label is shown for identical guide sequences from two different studies. A change from four to five allowed mismatches used in the scoring results in a change of the specificity scores but only in minor changes of the ranking of the guide sequences by specificity score. (PDF 23 kb) Additional file 8: Table S4. Comparison of the off-target prediction sensitivity of the CRISPR Design website (http://crispr.mit.edu/), CRISPOR, and CasOffFinder by the number of mismatches. Only off-targets with the PAM NGG were taken into account as the non-standard PAMs differ among the tools. Shown are the number of mismatches in the first columns, then over three columns the number of off-targets predicted for each tool, then two columns with the number of sites found by CRISPOR and CasOffFinder but not by the MIT site, then two columns with the number of sites found by the MIT site but not the other two tools (always 0). The next column shows an example sequence that was missed by the MIT site. The final column contains the distribution of mismatches for each nucleotide over the length of the guide sequence, for all sequences missed by the MIT site. (XLS 30 kb) Additional file 9: Figure S4. Similar to Fig. 3 but using off-target predictions by the CRISPR Design website (http://crispr.mit.edu/). **a** For the 28 tested guide sequences, MIT guide specificity scores as calculated by the MIT website (*x-axis*), number of off-targets (*y-axis*), and sum of off-target modification frequencies (*circle size*). **b** The specificity of the 28 tested guide sequences (*blue*) versus the specificity scores of all unique 20mers in human coding regions (*green*). The specificity score histogram was calculated by running 1000 randomly selected guide sequences from human coding regions through the CRISPR Design website (http://crispr.mit.edu/). (PDF 40 kb) Additional file 10: Table S5. Detailed information on CRISPR/Cas9 cleavage efficiency studies. Number of guides, cell types, delivery, and expression method of the guide RNA and comments. (XLS 36 kb) Additional file 11: Table S6. Correlations between knock-out efficiency results in different studies and cell types. (XLS 40 kb) Additional file 12: Collection of guide sequences and their frequencies from all published cleavage efficiency studies, including this one. The fields of the table are: the name of the study, the guide name (*guide*) and its sequence (*seq*), its extended sequence context (*longSeq*), the genome database used (*db*), the chromosomal position (*0-based*, *half-open*), the cleavage frequency (*modFreq*) reported by the study, and all scores calculated from the extended sequence, e.g., the Wang et al. score, the Chari et al. score, etc. (PNG 4950 kb) Additional file 13: Figure S5. Extended version of Fig. 4 *.* This figure includes the datasets not shown in Fig. 4. Shown are: Wang 2015 data, both human and mouse data from Doench 2014, Doench 2016, both cell lines tested by Chari et al., Housden score and Housden dataset, Liu dataset and the score-like efficiency heuristics from Ren et al. and Farboud et al. Labeling is similar to Fig. 4: datasets are indicated along the *y-axis* and scores for predicting guide actvity along the *x-axis*. Data points where the training data of the algorithm has been processed with the algorithm, so likely affected by over-fitting, are shown in *grey*. (PDF 22 kb) Additional file 14: Scatterplots of predicted versus obtained cleavage efficiency for all studies shown in Figs. 4 and 5 and Additional file 13: Figure S5 for all scoring models, including 3′ GC content-related heuristics. One row per dataset, one column per prediction score. The previously published datasets shown are, in order: the training data from the Wang, Doench 2014 (human and mouse) and Chari (293 T and K562) studies, Wang 2015, Doench 2016 (AZD and PLX treatment), Hart 2016 averaged over all time points (Rpe1 and the two Hct116 replicates), the data from studies by Moreno-Mateos, Varshney, Gagnon, Liu, Ren, Housden, Farboud, and Gandhi. For details and references on these datasets, see “Methods” or Additional file 10: Table S5. The last six rows represent datasets from this study: “Concordet” is a cell culture dataset quantified with T7 endonuclease and on gels, the datasets “Schönig” and “Eschstruth” ranked guides 1–3 by efficiency, “Shkumatava” by number of mutated sequencing clones obtained from zebrafish embryos, “Teboul” the percentage of mutant mouse embryos. For details on these datasets, see the “Methods” section and also Additional file 15: Table S7 for the Teboul dataset. (TSV 8773 kb) Additional file 15: Table S7. Details of the Teboul mouse dataset of this study. (XLS 70 kb) Additional file 16: Figure S6. Precision/recall curves for the large-scale datasets. The precision/recall plot for the Wong score on the Chari dataset looks different to that in the Wong et al. article as our study is analyzing only full datasets and Wong et al. used cross-fold data. The datasets by Hart, Doench 2016, Koike-Yusa, or Wang/Xu give the best impression of the Wong score on independent data that was not used for training by Wong et al. (PDF 87 kb) Additional file 17: Figure S7. Precision/recall for the top quartile against the top quartile of the predictions. Positives are the top 25 % of each assay. Precision is defined as the ratio True positives/(True positives + False positives) or intuitively the ability of a score not to label as positive a sample that is negative. The recall is the ratio True positives/(True positives + False negatives) or intuitively the ability of a score to find all the positive samples. When the cost of the assay is high and there are many candidate guide sequences, e.g., for a long exon in rats, the priority may be on precision or, conversely, it may be on recall for a short exon in *Drosophila*. Data are separated into three parts: (1) U6-base data, (2) T7 in vitro data, (3) data from this study. The rules by Ren and Farboud are already binary; all other scores were considered a positive if the rank-percent of the score exceeded 75 %. The 75 rank-percent cutoffs were: Housden, 6.8; Wang, 78; Chari, 53; Doench, 32; Moreno-Mateos, 60; Xu, 42; Fusi, 62; Wong, 0. The 75 % cutoff for the Wong score is indeed 0 as most values of this score are 0. The Wong score has a good precision on the large U6-based datasets but a relatively low recall. Among the heuristics, the -GG rule from Farboud et al. has high precision for T7 in vitro transcription datasets, except for the Farboud dataset where some guides have been designed to fulfill the rule, so it is not unbiased. In U6-based datasets, most scores show a similar precision, but the Fusi et al. score has generally higher recall. (PDF 147 kb)
