# Supplementary figures and images for: Evaluation of off-target and on-target scoring algorithms and integration into the guide RNA selection tool CRISPOR
Source: Genome Biol. 2016 Jul 5;17:148. doi: 10.1186/s13059-016-1012-2 (PMC4934014; doi:10.1186/s13059-016-1012-2)

## EMX1

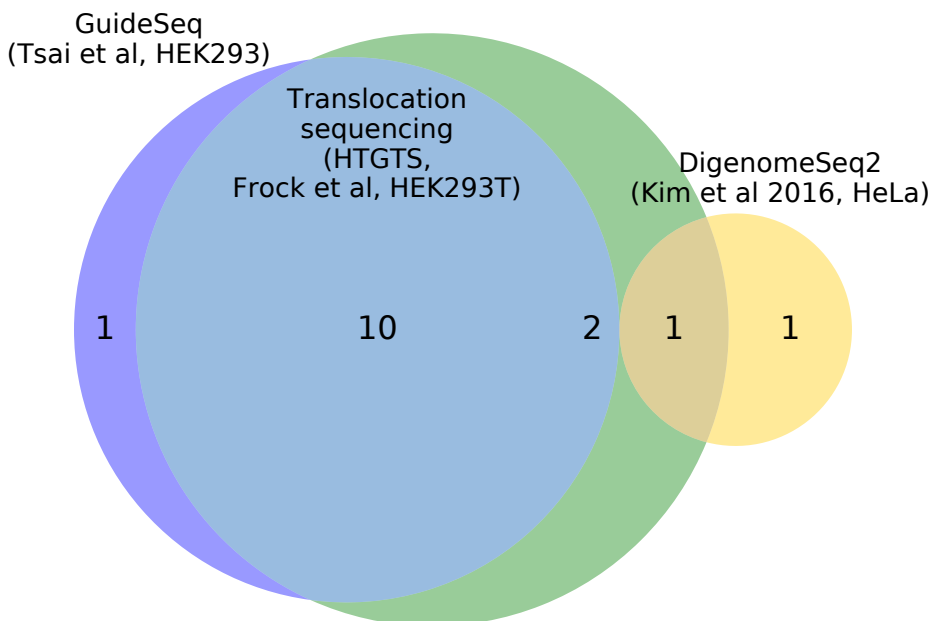

## VEGFA site1

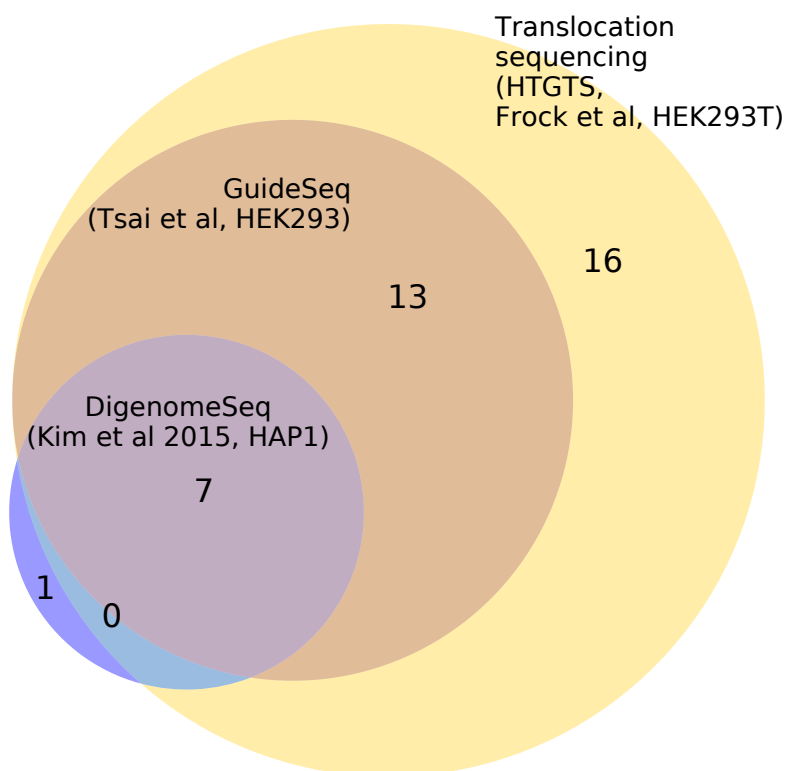

Supplement: Additional file 3: Figure S1. — Overlap of off-target detection for the EMX1 and VEGFA guides tested by different assays. Off-targets are only shown if they were detected by at least a single study and with a frequency of 0.1 %. See Additional file 1: Tables S1 and Additional file 4: Table S2 for the modification frequencies and additional details on the off-targets for the guides EMX1 and VEGFA, respectively. Additional file 4: Table S2 also includes the data by Hsu et al. [7], who quantified cleavage at putative off-target loci predicted by the CRISPR Design website (http://crispr.mit.edu/) with targeted deep sequencing, Tsai et al. [3], who isolated double-strand breaks with modified oligonucleotides followed by sequencing, Frock et al. [28], who detected translocations, and Kim et al. [33] and Kim et al. [27], who performed whole-genome sequencing to find CRISPR-induced modifications. For details on the different studies, see Additional file 1: Table S1. (PDF 17 kb) [file 13059_2016_1012_MOESM3_ESM.pdf]

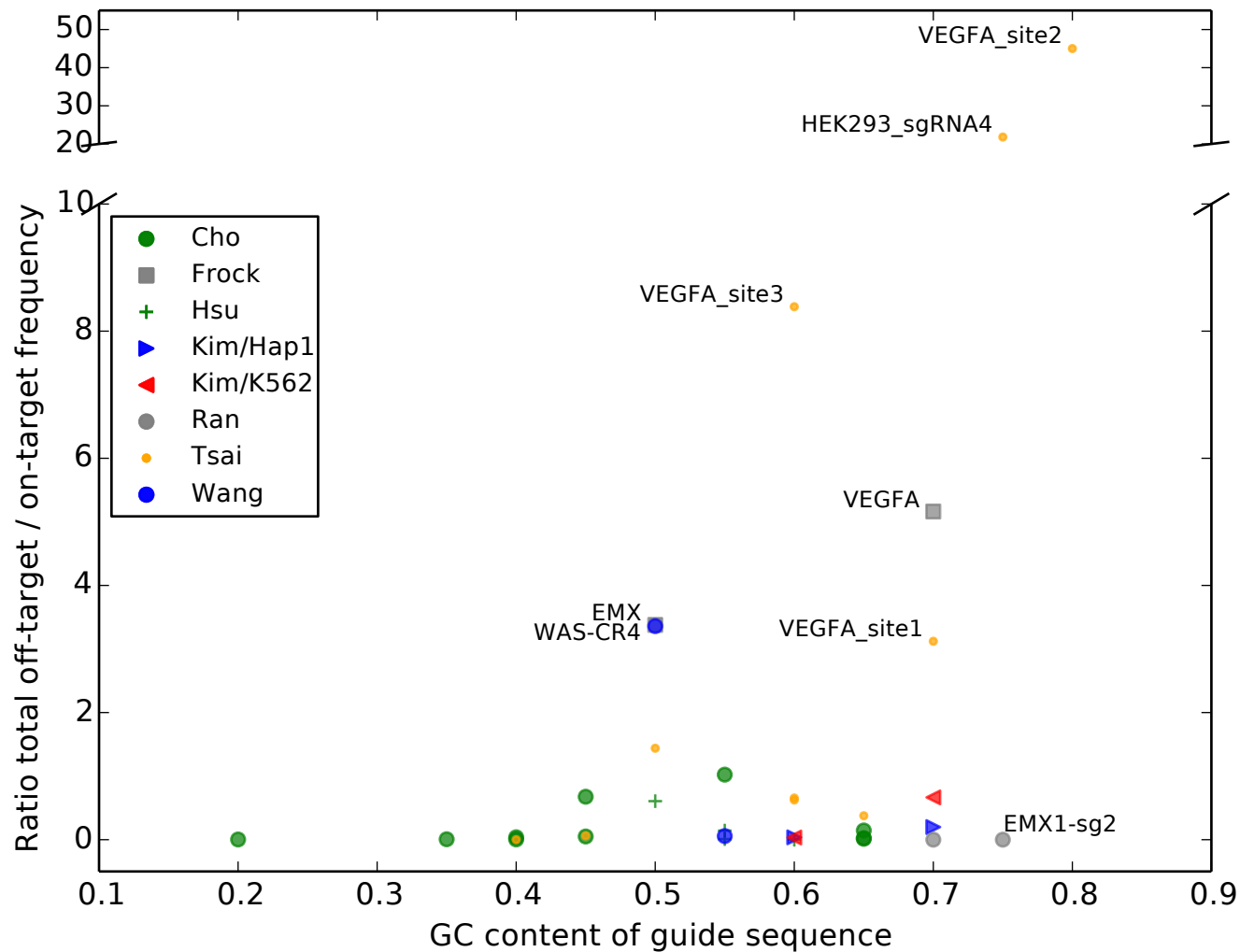

Supplement: Additional file 6: Figure S2. — Ratio of off-target to on-target cleavage for validated guide sequences. The two guides RAG1B and RAG1A are not shown on this plot as their on-target cleavage was not determined in the study by Frock et al. [28]. Studies in the legend are referenced by the first author’s name; in the case of Kim et al., the cell type is also indicated. For each guide, the sum of all off-target modification frequencies was divided by the on-target modification frequency, e.g., a ratio of 2 indicates that cleavage is twice as frequent on all off-targets taken together than on the target. To better show the two outliers, a portion of the x-axis and y-axis was cut out. The CRISPOR website and Genome Browser tracks show a warning message for guide sequences with a GC content >75 %. (PDF 66 kb) [file 13059_2016_1012_MOESM6_ESM.pdf]

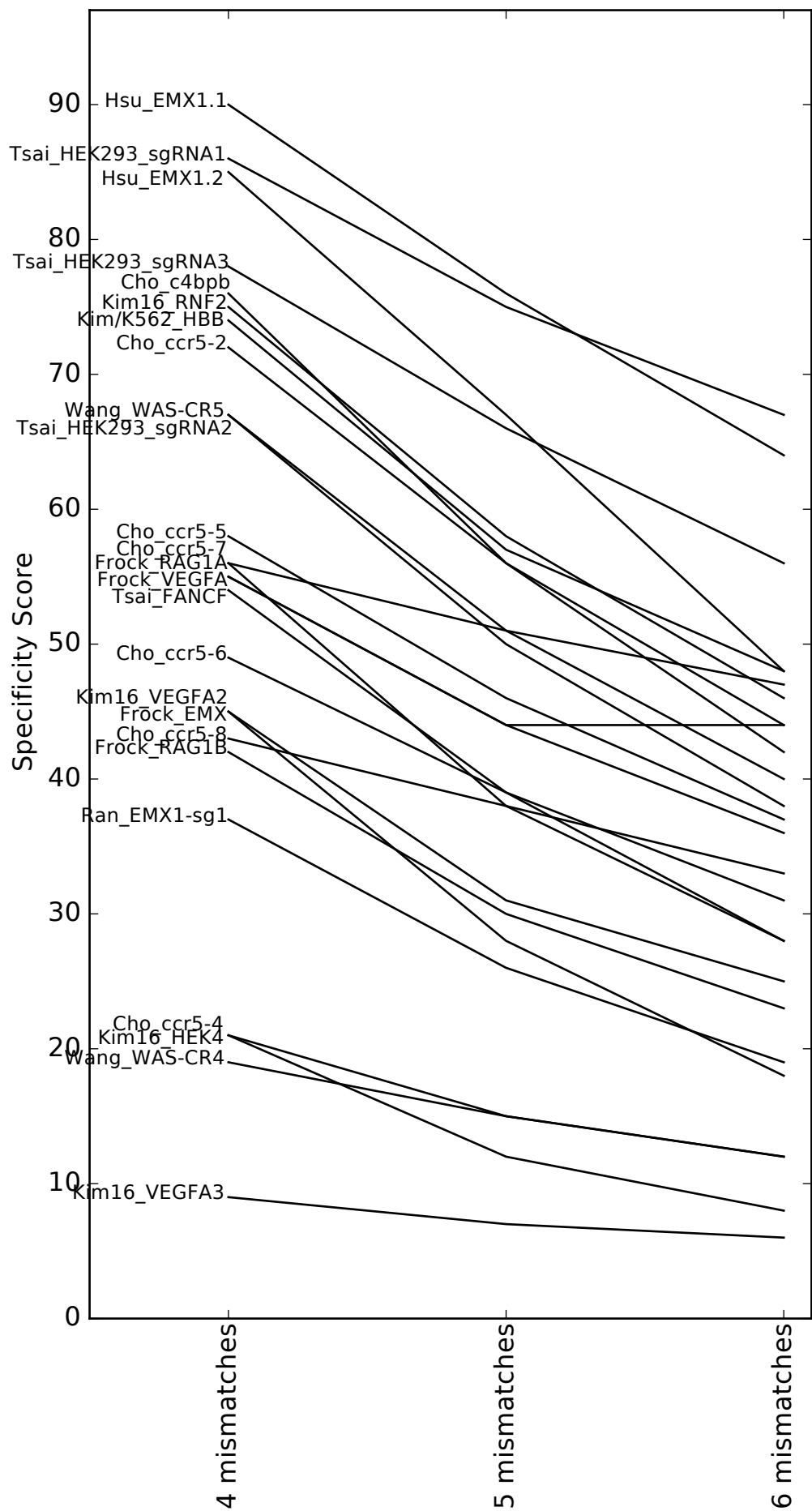

Supplement: Additional file 7: Figure S3. — MIT specificity scores calculated by the CRISPOR website for 28 guide sequences calculated based on predicted off-targets with up to four, five, and six mismatches. Only one label is shown for identical guide sequences from two different studies. A change from four to five allowed mismatches used in the scoring results in a change of the specificity scores but only in minor changes of the ranking of the guide sequences by specificity score. (PDF 23 kb) [file 13059_2016_1012_MOESM7_ESM.pdf]

A

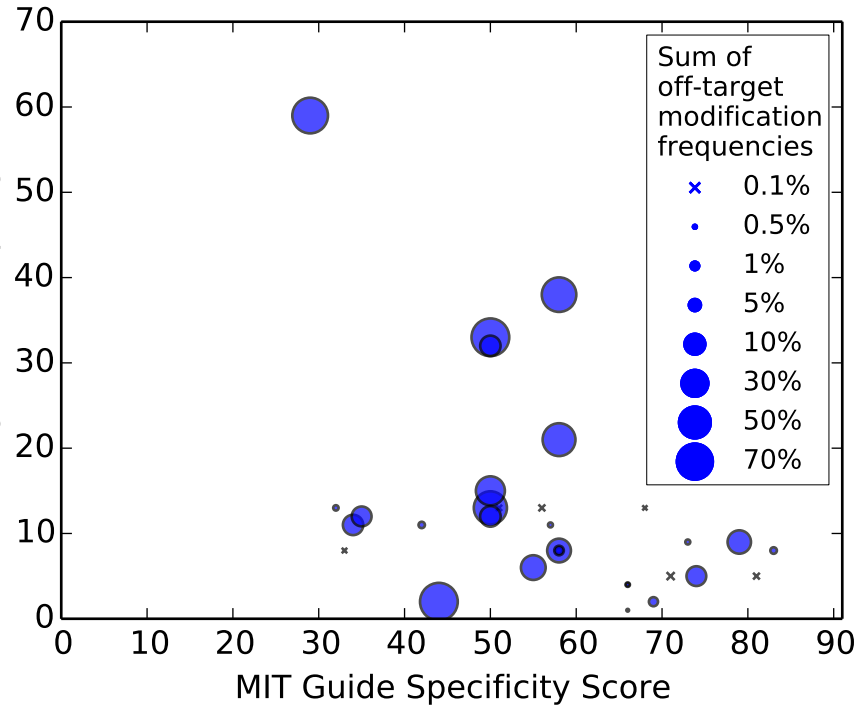

B

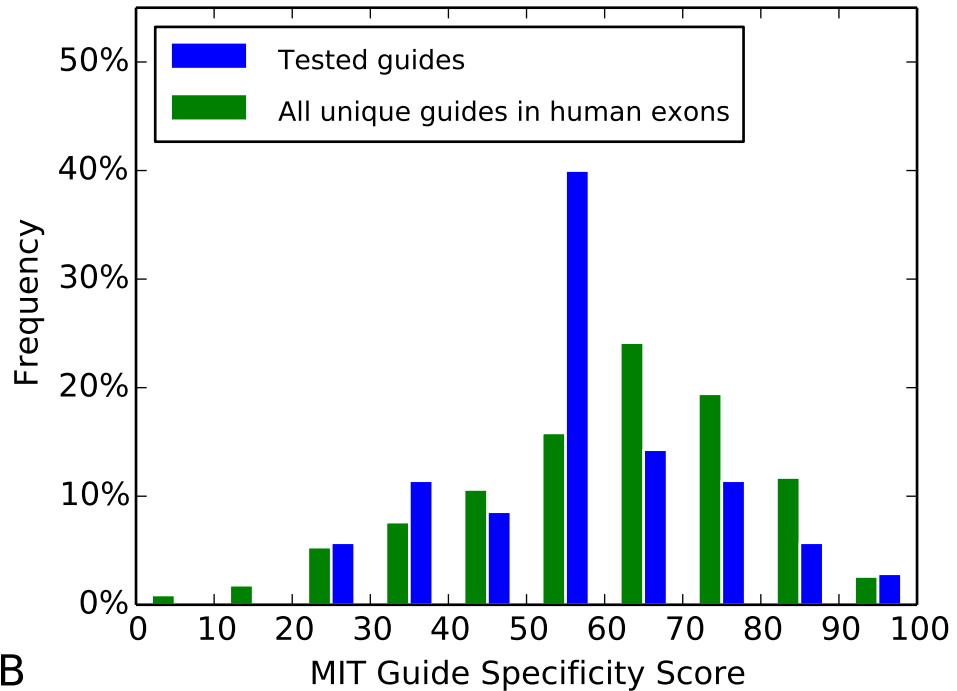

Supplement: Additional file 9: Figure S4. — Similar to Fig. 3 but using off-target predictions by the CRISPR Design website (http://crispr.mit.edu/). a For the 28 tested guide sequences, MIT guide specificity scores as calculated by the MIT website (x-axis), number of off-targets (y-axis), and sum of off-target modification frequencies (circle size). b The specificity of the 28 tested guide sequences (blue) versus the specificity scores of all unique 20mers in human coding regions (green). The specificity score histogram was calculated by running 1000 randomly selected guide sequences from human coding regions through the CRISPR Design website (http://crispr.mit.edu/). (PDF 40 kb) [file 13059_2016_1012_MOESM9_ESM.pdf]

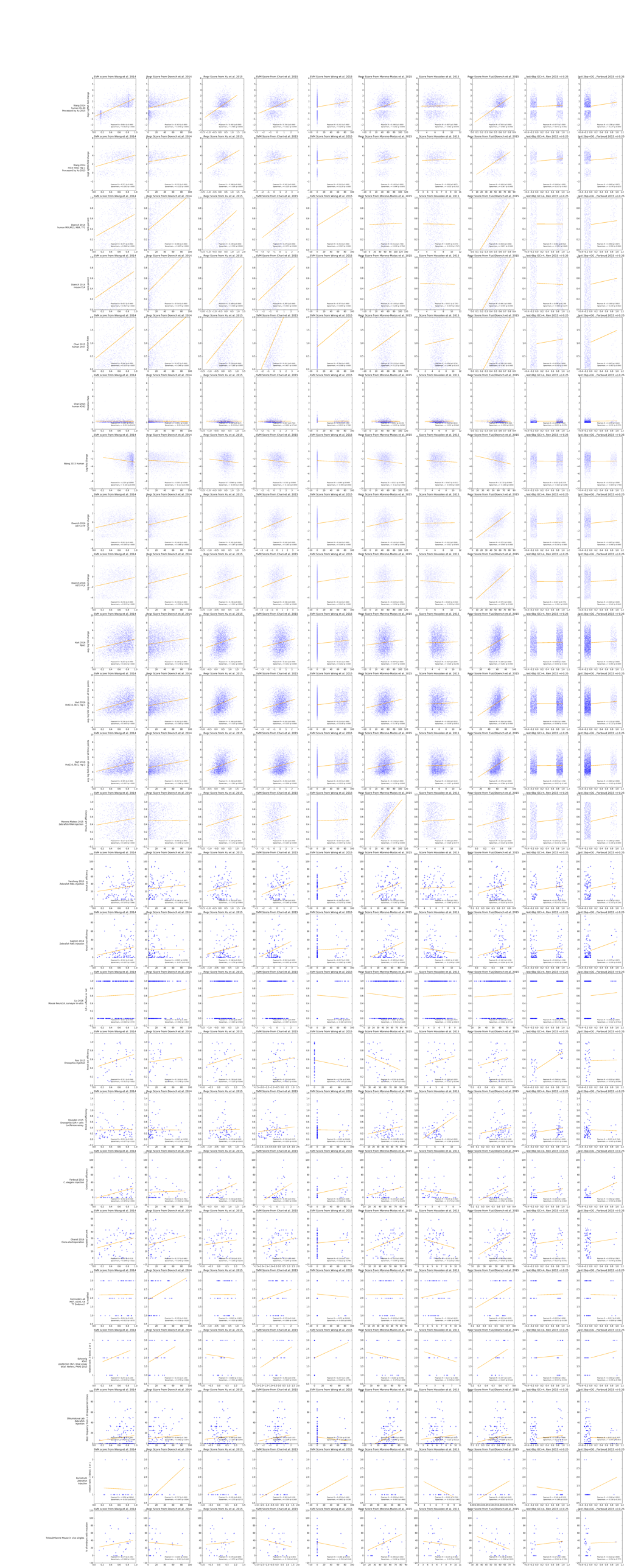

Supplement: Additional file 12: — Collection of guide sequences and their frequencies from all published cleavage efficiency studies, including this one. The fields of the table are: the name of the study, the guide name (guide) and its sequence (seq), its extended sequence context (longSeq), the genome database used (db), the chromosomal position (0-based, half-open), the cleavage frequency (modFreq) reported by the study, and all scores calculated from the extended sequence, e.g., the Wang et al. score, the Chari et al. score, etc. (PNG 4950 kb) [file 13059_2016_1012_MOESM12_ESM.png]

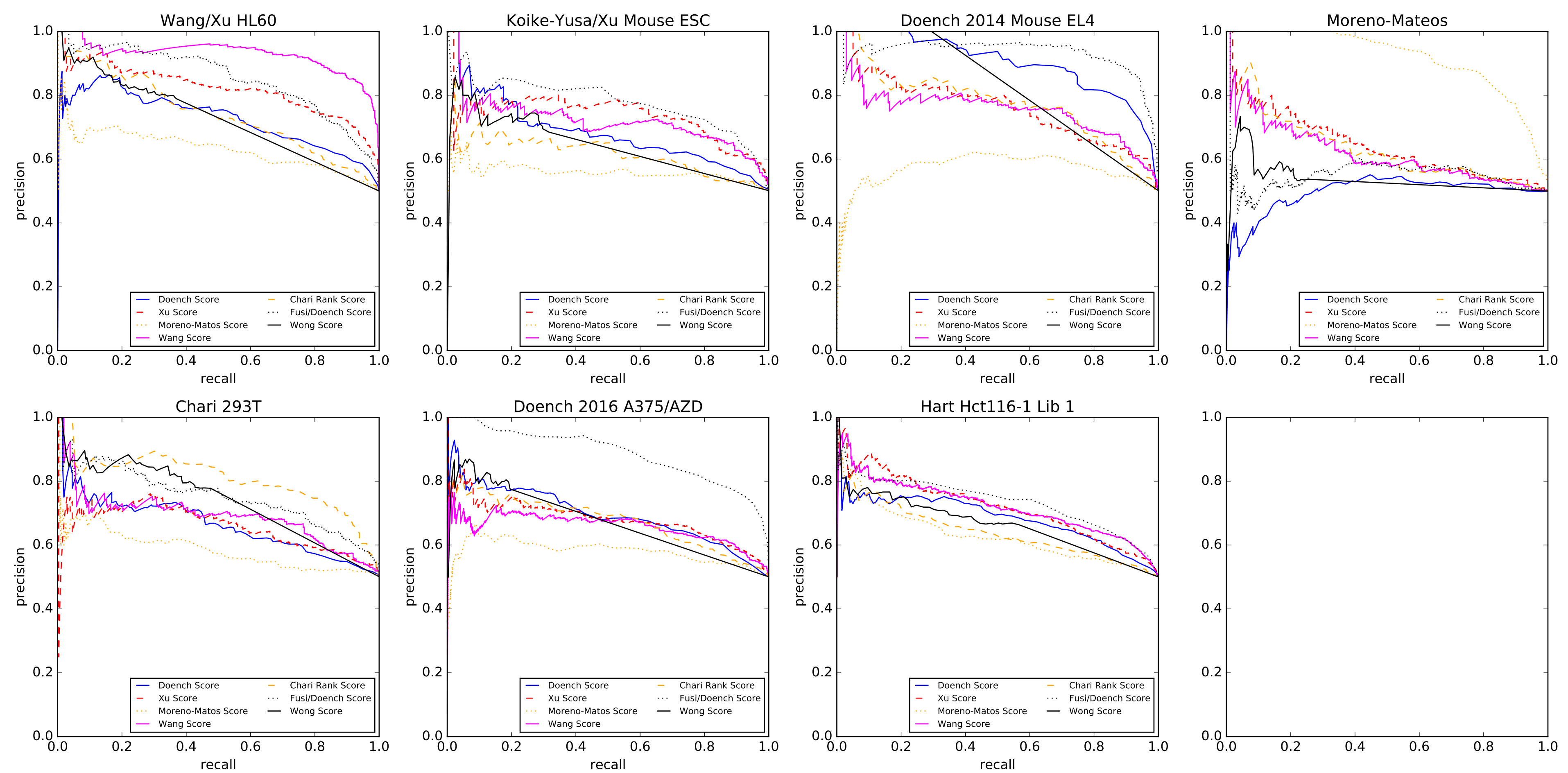

Supplement: Additional file 16: Figure S6. — Precision/recall curves for the large-scale datasets. The precision/recall plot for the Wong score on the Chari dataset looks different to that in the Wong et al. article as our study is analyzing only full datasets and Wong et al. used cross-fold data. The datasets by Hart, Doench 2016, Koike-Yusa, or Wang/Xu give the best impression of the Wong score on independent data that was not used for training by Wong et al. (PDF 87 kb) [file 13059_2016_1012_MOESM16_ESM.pdf]
