# Supplementary material for: Evaluation of off-target and on-target scoring algorithms and integration into the guide RNA selection tool CRISPOR
Source: Genome Biol. 2016 Jul 5;17:148. doi: 10.1186/s13059-016-1012-2 (PMC4934014; doi:10.1186/s13059-016-1012-2)
Supplement: Additional file 13: Figure S5. — Extended version of Fig. 4 . This figure includes the datasets not shown in Fig. 4. Shown are: Wang 2015 data, both human and mouse data from Doench 2014, Doench 2016, both cell lines tested by Chari et al., Housden score and Housden dataset, Liu dataset and the score-like efficiency heuristics from Ren et al. and Farboud et al. Labeling is similar to Fig. 4: datasets are indicated along the y-axis and scores for predicting guide actvity along the x-axis. Data points where the training data of the algorithm has been processed with the algorithm, so likely affected by over-fitting, are shown in grey. (PDF 22 kb) [file 13059_2016_1012_MOESM13_ESM.pdf]

	Wang Score	Doench Score	Xu Score	Chari Score	Wong Score	Moreno–Matos Score	Housden Score	Fusi/Doench Score	Ren: last 6 bp GC>4	Farboud: ends with GG
Wang/Xu HL60 (2076)	0.616	0.343	0.486	0.321	0.246	0.201	0.002	0.485	0.071	0.137
Wang 2015 using Wang Score (2921)	−0.104	−0.11	−0.085	−0.102	−0.094	−0.114	−0.04	−0.168	−0.024	−0.005
Doench 2014 MOLM13/NB4/TF1 (881)	0.366	0.497	0.33	0.273	0.307	0.01	−0.012	0.657	−0.002	0.09
Doench 2014 Mouse EL4 (951)	0.427	0.577	0.4	0.403	0.369	0.156	−0.007	0.7	−0.048	0.1
Doench 2016 A375/AZD (2333)	0.265	0.266	0.287	0.245	0.164	0.144	0.012	0.54	0.104	0.09
Koike−Yusa/Xu 1 Mouse ESC (907)	0.281	0.221	0.306	0.12	0.119	0.094	0.032	0.367	0.103	0.074
Chari 293T (1234)	0.31	0.246	0.286	0.457	0.308	0.123	0.046	0.381	0.1	0.083
Chari K562 (1239)	−0.013	−0.019	−0.019	0.009	0.011	0.013	0.032	−0.035	0.051	0.031
Hart Rpe (4214)	0.232	0.178	0.201	0.152	0.162	0.077	0.018	0.281	0.04	0.065
Hart Hct116−1 Lib 1 (4293)	0.335	0.285	0.31	0.21	0.228	0.176	0.03	0.369	0.008	0.122
Hart Hct116−2 Lib 1 (4239)	0.307	0.288	0.292	0.208	0.232	0.159	0.027	0.384	0.023	0.093
Farboud C. elegans (50)	0.476	0.301	0.545	0.602	0.4	0.177	−0.171	0.541	0.431	0.593
Ren Drosophila (39)	0.313	0.178	0.225	0.152	−0.158	−0.347	0.212	0.131	0.811	0.034
Liu Neuro2A Surveyor 1/0 (205)	0.04	0.091	0.063	0.007	−0.042	−0.054	0.014	0.044	0.123	0.042
Eschstruth Zebrafish (18)	−0.014	0.647	0.273	0.33	0.491	−0.043	−0.331	0.503	−0.149	0.316
Housden Dros−S2R+ (75)	0.077	0.137	0.124	0.25	0.024	0.204	0.394	0.251	0.331	0.005
Varshney (102)	0.17	0.139	0.171	0.28	0.27	0.262	0.066	0.219	0.21	0.153
Gagnon (111)	0.207	−0.072	0.179	0.202	0.083	0.357	−0.134	0.104	0.162	0.209
Moreno−Mateos (1020)	0.14	0.038	0.171	0.145	0.037	0.579	0.028	0.12	0.14	0.18
Teboul Mouse In Vivo (30)	0.188	−0.083	0.072	−0.154	−0.219	0.426	−0.017	−0.304	0.182	0.054
Schoenig K562 LacZ Rank (24)	0.219	0.273	0.011	0.42	0.283	−0.178	−0.236	0.415	0.103	0.205
Shkumatava Zebrafish (162)	0.14	0.081	0.067	0.135	−0.125	0.258	0.125	0.108	0.125	0.004
