# Supplementary material for: Evaluation of off-target and on-target scoring algorithms and integration into the guide RNA selection tool CRISPOR
Source: Genome Biol. 2016 Jul 5;17:148. doi: 10.1186/s13059-016-1012-2 (PMC4934014; doi:10.1186/s13059-016-1012-2)
Supplement: Additional file 17: Figure S7. — Precision/recall for the top quartile against the top quartile of the predictions. Positives are the top 25 % of each assay. Precision is defined as the ratio True positives/(True positives + False positives) or intuitively the ability of a score not to label as positive a sample that is negative. The recall is the ratio True positives/(True positives + False negatives) or intuitively the ability of a score to find all the positive samples. When the cost of the assay is high and there are many candidate guide sequences, e.g., for a long exon in rats, the priority may be on precision or, conversely, it may be on recall for a short exon in Drosophila. Data are separated into three parts: (1) U6-base data, (2) T7 in vitro data, (3) data from this study. The rules by Ren and Farboud are already binary; all other scores were considered a positive if the rank-percent of the score exceeded 75 %. The 75 rank-percent cutoffs were: Housden, 6.8; Wang, 78; Chari, 53; Doench, 32; Moreno-Mateos, 60; Xu, 42; Fusi, 62; Wong, 0. The 75 % cutoff for the Wong score is indeed 0 as most values of this score are 0. The Wong score has a good precision on the large U6-based datasets but a relatively low recall. Among the heuristics, the -GG rule from Farboud et al. has high precision for T7 in vitro transcription datasets, except for the Farboud dataset where some guides have been designed to fulfill the rule, so it is not unbiased. In U6-based datasets, most scores show a similar precision, but the Fusi et al. score has generally higher recall. (PDF 147 kb) [file 13059_2016_1012_MOESM17_ESM.pdf]

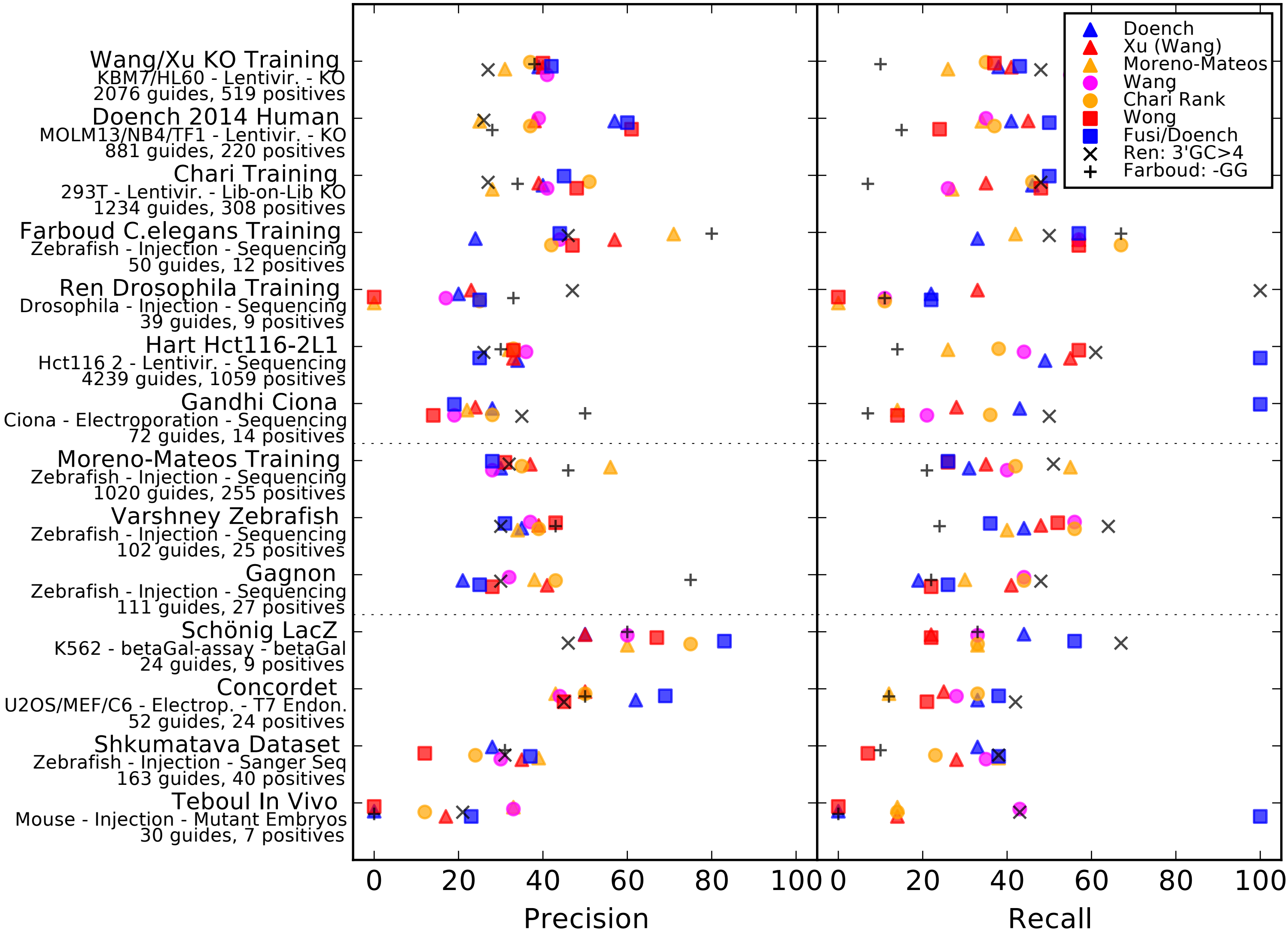

For the dataset Concordet, the top 25 % of the guides are not one-quarter of the data points, as the assay result was rank-transformed and the top rank covers 45 % of the guides. For the sake of completeness we are showing all datasets, even though we note that for smaller ones the results are based on very small numbers of instances and should be considered with caution, e.g., for the -GG rule, only one-sixteenth of guides fulfill this rule on average. By their definition, the scores by Xu and Fusi are trained on the datasets by Wang and Doench, respectively, which is highlighted in parentheses in the legend. Regression-based models are shown as triangles, support vector machine (SVM)-based ones as circles, and the rules from Ren and Farboud as crosses. Squares indicate other types of models: the Fusi score is a gradient-boosted regression tree and the Housden score is the similarity to a weight matrix. Linear regression prediction models are shown with triangles, SVM-based ones with circles. Crosses indicate heuristic rules. Scoring models are named according to the first author of the respective study. Overall, scores usually perform best on their own training dataset.
